# Identification of Causative Ciguatoxins in Red Snappers *Lutjanus bohar* Implicated in Ciguatera Fish Poisonings in Vietnam

**DOI:** 10.3390/toxins10100420

**Published:** 2018-10-20

**Authors:** Dao Viet Ha, Aya Uesugi, Hajime Uchida, Pham Xuan Ky, Dang Quoc Minh, Ryuichi Watanabe, Ryoji Matsushima, Hiroshi Oikawa, Satoshi Nagai, Mitsunori Iwataki, Yasuwo Fukuyo, Toshiyuki Suzuki

**Affiliations:** 1 Institute of Oceanography, Vietnam Academy of Science and Technology, 01 Cau Da, Nha Trang 650000, Vietnam; dvha@vast.org.vn (D.V.H.); phamkx@vnio.org.vn (P.X.K.); qminh2005@yahoo.com (D.Q.M.); 2 National Research Institute of Fisheries Science, 2-12-4 Fukuura, Kanazawa-ku, Yokohama 236-8648, Japan; auesugi@affrc.go.jp (A.U.); huchida@affrc.go.jp (H.U.); rwatanabe@affrc.go.jp (R.W.); matsur@affrc.go.jp (R.M.); oikawah@fra.affrc.go.jp (H.O.); snagai@affrc.go.jp (S.N.); 3 Asian Natural Environmental Science Center, University of Tokyo, 1-1-1 Yayoi, Bunkyo, Tokyo 113-8657, Japan; iwataki@anesc.u-tokyo.ac.jp (M.I.); ufukuyo@mail.ecc.u-tokyo.ac.jp (Y.F.)

**Keywords:** ciguatera fish poisoning (CFP), ciguatoxin-1B (CTX-1B), LC/MS, red snapper, TOF, Vietnam

## Abstract

Ciguatera fish poisoning (CFP) is a type of food poisoning caused by the consumption of a variety of toxic ciguatera fish species in the tropical and subtropical waters. Although there have been a large number of suspected CFP cases in the Southeast Asian countries, few were confirmed with causative ciguatoxins (CTXs), and reliable information on the symptoms still remains rather limited. In the present study, CTXs in red snapper *Lutjanus bohar*, implicated in two suspected CFP cases in Vietnam in 2014 and 2016, were determined by use of the single-quadrupole selected ion monitoring (SIM) liquid chromatography/mass spectrometry (LC/MS). Ciguatoxin-1B (CTX-1B), 54-deoxyCTX-1B, and 52-*epi*-54-deoxyCTX-1B were detected in the red snapper by our LC/MS method. Moreover, CTX-1B, 54-deoxyCTX-1B, and 52-*epi*-54-deoxyCTX-1B were further identified by the time of flight (TOF) LC/MS with the exact mass spectrum. The CTX profile of the red snapper in Vietnam is similar to those of ciguatera fish from Australia, Okinawa Islands in Japan, Kiribati, and Hong Kong. This is the first comprehensive report unambiguously identifying the causative toxins in fish implicated with reliable information on the poisoning symptoms in CFP in Vietnam and/or Southeast Asian countries.

## 1. Introduction

Ciguatera fish poisoning (CFP) is a type of seafood poisoning caused by the consumption of tropical and subtropical fish whose flesh is contaminated with lipophilic polyether ciguatoxin (CTX) and its analogues. CFP is considered as one of the largest scale food poisoning of nonbacterial origin in the world, with at least more than ten thousand people estimated to suffer from it annually [1]. CFP symptoms include neurological (e.g., tingling, itching, a disorder of temperature sensation), gastrointestinal (e.g., vomiting, diarrhea, nausea), and cardiovascular (e.g., hypotension, bradycardia) disorders in humans, which may last up to one month or more [1,2,3]. Ciguatoxins (CTXs) were first reported to be produced by a certain strain of the dinoflagellate *Gambierdiscus toxicus* [4,5,6]. Recent research has shown that *G. polynesiensis* and *G. excentricus* are also primary toxin-producing species [7,8,9]. CTXs are accumulated in various kinds of reef fish and marine invertebrates [10,11,12] through the food chain. CTXs are classified as Pacific (P) [13,14], Caribbean (C) [15], and Indian Ocean (I) CTX-group toxins, based on their chemical structures. The different CTXs may cause a variety of poisoning symptoms [2]; for example, neurological symptoms predominate in CFP cases in the Pacific Ocean, while gastrointestinal symptoms are the dominant feature in CFP cases in the Caribbean Ocean [2,3]. There is no regulatory limit for CTX in fish, but a guidance level of 0.01 ppb CTX-1B equivalents, based on a 10-fold reduction of the lowest concentration of CTX in meal remnants found to cause human illness [16], has been set by the United States Food and Drug Administration (US FDA).

Although a large number of CFP cases were reported sporadically in the Southeast Asian countries [17], little is known on the causative ciguatoxins (CTXs) in implicated fish, and reliable information on the poisoning symptoms remains very limited. Since 2007, suspected CFP cases by the consumption of red snappers have been increasingly reported in Vietnam [18]. Also, at least five *Gambierdiscus* species are commonly found in Vietnamese coastal waters [19]. In our previous study [20], a LC/MS method was developed using a red snapper implicated in a CFP incident in Vietnam in 2014, however, quantification of the causative toxins was not carried out, due to a lack of reliable reference material. Moreover, detailed symptoms of the CFP incident were not reported in the previous study [20]. In the present study, the red snapper specimen involved in the 2014 poisoning incident was re-analyzed to quantify the causative toxin with a reliable reference material. Another red snapper, implicated in a suspected CFP incident in 2016, was also investigated. This is the first comprehensive report on CFP incidents in Vietnam that include identification and quantification of the causative toxins associated with detailed symptoms.

## 2. Results

Table 1 summarizes two CFP incidents that occurred in Vietnam in 2014 (referred to as incident 1) and 2016 (referred to as incident 2). Typical gastrointestinal and neurological symptoms of CFP were observed in the patients from both the incidents. On the other hand, neurological symptoms, which invariably occur in CFP in the Pacific Ocean [2,3]; especially, unusual temperature perception disturbances were reported in one of the patients from incident 2. From the reported symptoms, both incident 1 and 2 were characterized as cases of CFP. The red snapper specimen implicated in CFP incident 1 was identified as *Lutjanus bohar* by 16S rDNA and COX gene analysis. Although genetic analysis of the red snapper specimen from incident 2 was not carried out, this red snapper was unambiguously identified as *Lutjanus bohar* on the basis of its morphology.

During optimization of MS parameters, we found that the declustering potential voltage (DP) and the ion source temperature (TEM) were the most important parameters affecting the peak intensities on the single-quadrupole selected ion monitoring (SIM) LC/MS chromatograms. Higher peak intensities were obtained with higher DP and TEM.

**Figure 1:**
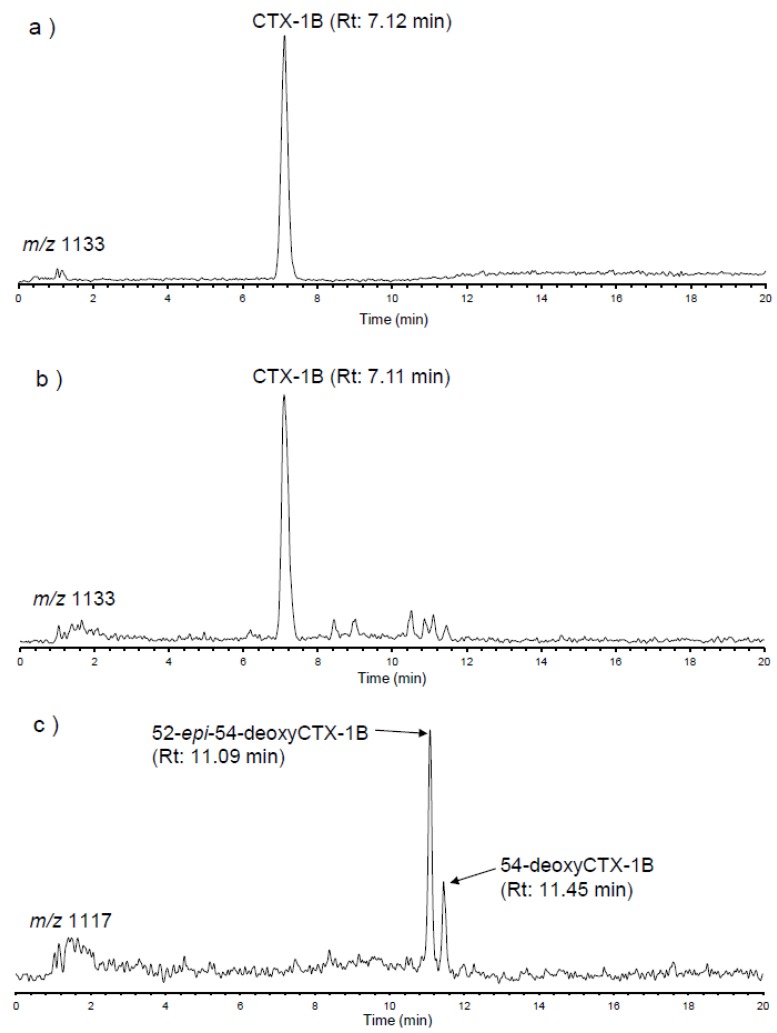
The SIM LC/MS chromatogram for [M+Na]^+^ of ciguatoxins (CTXs) obtained from the red snapper implicated in CFP in Vietnam: (**a**) Authentic CTX-1B; (**b**) CTX-1B in red snapper; (**c**) 52-*epi*-54-deoxy CTX-1B and 54-deoxy CTX-1B in red snapper. Rt: Retention time.

**Figure 2:**
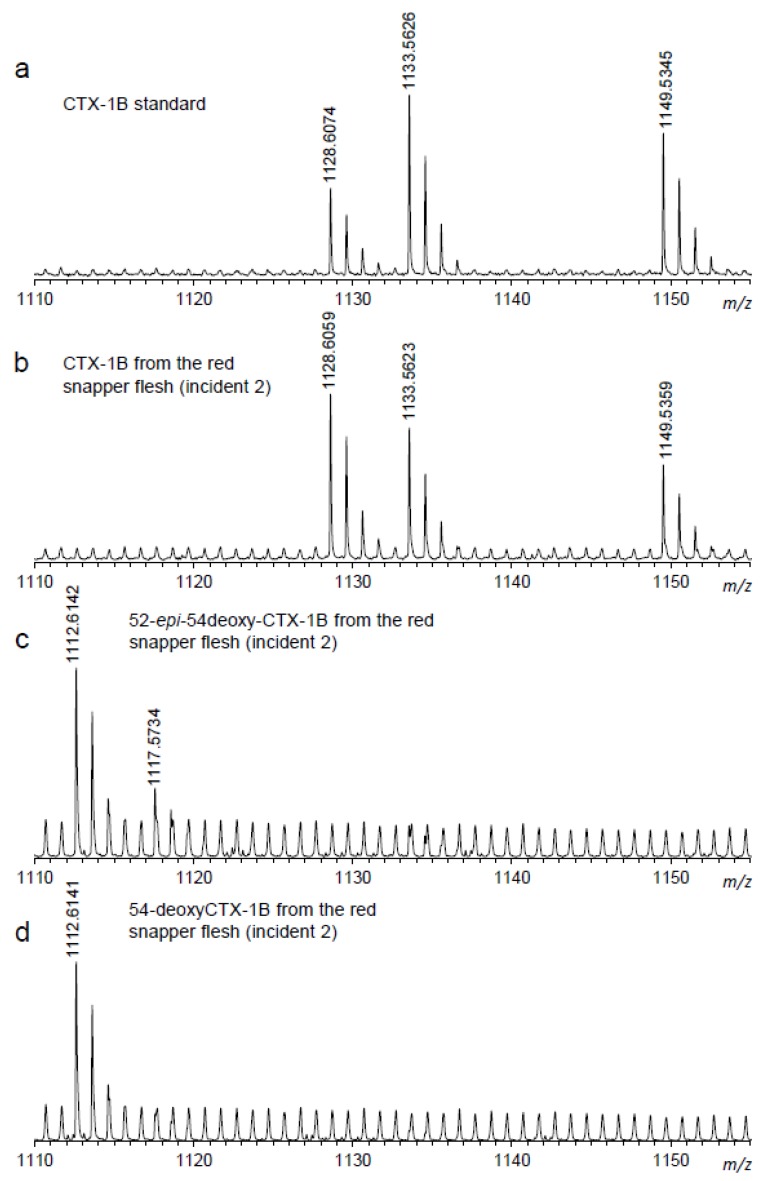
High resolution mass spectra of CTX-1B and 54-deoxyCTX-1B obtained from the red snapper implicated in CFP in Vietnam. (**a**): CTX-1B standard; (**b**): CTX-1B from red snapper flesh (incident 2); (**c**): 52-*epi*-54-deoxyCTX-1B from red snapper flesh (incident 2); (**d**): 54-deoxyCTX-1B from red snapper flesh (incident 2).

The SIM LC/MS chromatograms of authentic CTX-1B and the extract of the red snapper implicated in the CFP incident 1 are shown in Figure 1. The peak with the same retention time as authentic CTX-1B was detected in the red snapper extract. Further confirmation of CTX-1B, detected in the red snapper specimens, was carried out by measuring high resolution mass spectra (HRMS) on the time of flight (TOF) LC/MS. Table 2 lists the theoretical and the measured values of HRMS corresponding to observed positive adduct ions of CTX-1B and its deoxy analogues. The measured values were well matched with the theoretical values of [M+NH_4_]^+^, [M+Na]^+^ and [M+K]^+^ of CTX-1B, within 10 ppm tolerance. The isotopic patterns of each adduct ion of CTX-1B, obtained from the red snapper specimens, were also close to that from authentic CTX-1B (Figure 2). Peaks corresponding to 54-deoxyCTX-1B and 52-*epi*-54-deoxyCTX-1B, were also detected in the red snappers in Vietnam (Figure 1c). Identification of the peaks of 54-deoxyCTX-1B and 52-*epi*-54-deoxyCTX-1B was carried out by the elution order of epimers in a reversed phase chromatography reported in a previous study [21]. Although authentic 54-deoxyCTX-1B and 52-*epi*-54-deoxyCTX-1B were not available, HRMS obtained from the red snappers agreed with that of the theoretical values within 10 ppm tolerance (Table 2). In contrast to CTX-1B, ions corresponding to [M+Na]^+^ and [M+K]^+^ were hardly detected in other analogues, due to low concentrations of these toxins (Figure 2). Although all CTX analogues found in Pacific region were screened for in the present study, no CTX analogues other than CTX-1B and its deoxy analogues were detected. These results indicate that the dominant CTXs in the red snappers implicated in CFP in Vietnam were CTX-1B and its deoxy analogues. CTX was not detected in the retrospective analysis of mouse bioassay (MBA) negative coral reef fish specimens.

When using MS with electrospray ionization (ESI), there is competition (matrix effects) in the source between matrix components and analytes that results in ion enhancement or suppression of signal intensities. In the present study, intensities of SIM LC/MS signals for the [M+Na]^+^ ion of CTX-1B, obtained from red snapper matrices and methanol, were compared. Following a previous method [21], the concentration of CTX-1B in the sample solution was 22 ng/mL, corresponding to 0.88 ng/g fish flesh. The SIM LC/MS signal obtained in the presence of the matrix was 27% of that obtained from methanol solution. This result indicates that the red snapper matrix causes ion suppression in our SIM LC/MS for the [M+Na]^+^ ion of CTX-1B. Limits of detection (LOD) and quantification (LOQ), obtained from the red snapper fortified with CTX-1B at a concentration of 22 ng/mL, were 0.09 and 0.32 ng/g fish flesh, respectively.

The CTX concentrations, quantified by LC/MS in the red snappers implicated in the CFP cases in Vietnam, are listed in Table 3. CTX-1B, along with 54-deoxyCTX-1B and 52-*epi*-54-deoxyCTX-1B, were detected in the red snapper from incident 1. The CTX concentrations in Table 3 were all adjusted with respect to the results of the matrix effects experiment on the red snapper specimen. The CTX-1B content was significantly higher than that of 54-deoxyCTX-1B and 52-*epi*-54-deoxyCTX-1B. 54-deoxyCTX-1B and its epimer were not detected in the red snapper from incident 2, whereas trace levels of 52-*epi*-54-deoxyCTX-1B were detected in the same sample by TOF LC/MS. The concentrations of CTX-1B in the specimens from the incident 1 and 2 are between 0.9 and 3.7 ng/g fish flesh. The total mouse toxicity of the red snapper flesh from the incident 2, estimated by LC/MS, was close to that obtained by MBA, whereas the value from the incident 1 estimated by LC/MS was four times higher than that obtained by MBA.

## 3. Discussion

It is reported that neurological symptoms predominate in CFP cases in the Pacific Ocean, while gastrointestinal symptoms are the dominant feature in the Caribbean Ocean [2,3]. Symptoms reported in the patients in putative CFP cases in Vietnam were neurological, and coincided with typical symptoms reported from the Pacific Ocean. The presence of CTX-1B, along with 54-deoxyCTX-1B and 52-*epi*-54-deoxyCTX-1B, was unambiguously confirmed in red snappers implicated in the CFP in Vietnam by our SIM LC/MS and TOF LC/MS methods.

In previous studies, it was reported that P-CTX1 (CTX-1B), P-CTX2 (52-*epi*-54-deoxyCTX-1B), and P-CTX3 (54-deoxyCTX-1B) were the most commonly observed toxins found in fish implicated in CFP cases in Pacific Ocean areas [21,24,25,26]. Toxin profiles obtained from the red snappers implicated in CFP in Vietnam were similar to those reported in other areas in the Pacific Ocean, Okinawa Islands in Japan [21], Australia [24], Kiribati [25], and Hong Kong [26]. Although there have been large numbers of reported or potential CFP cases in the Southeast Asian countries, there are no reports identifying the causative CTXs in fish implicated in CFP cases that have reliable information on poisoning symptoms. Our findings are the first to comprehensively identify the causative CTXs in Southeast Asia, where CFPs are of importance in terms of the size of potentially affected populations and the growth of fisheries activities.

The CONTAM Panel of European Food Safety Authority (EFSA) noted that several publications state that cases of CFP in the Pacific mostly occur following the consumption of fish containing the equivalent of 0.1–5 ng CTX-1B (P-CTX-1)/g of fish flesh [27]. The concentrations of CTX-1B in red snappers quantified in the present study was 0.9–3.7 ng/g fish flesh (Table 3), which were within the range reported from previous CFP cases. The estimated dose that triggers mild ciguatera symptoms is reported as 1 ng CTX-1B/kg body weight (b.w.) [28]. Another report estimated that consumption of at least 70 ng of CTX-1B causes human poisoning [29]. Therefore, approximately 19–78 g fish flesh could be consumed by the patients in incidents 1 and 2 in Vietnam. Although the mouse toxicity of the fish flesh from incident 1, as estimated by LC/MS, was inconsistent with that obtained by the MBA, it could be explained by inaccuracy of the MBA. Noteworthily, the CTX-1B level in the fish flesh was higher than that in the fish viscera in incident 2 (Table 3). There is very little knowledge on the distribution of CTXs in fish in Vietnam, and further investigation is necessary.

The single SIM LC/MS method [20], modified from the quadrupole tandem LC/MS/MS method selecting [M+Na]^+^ ions of CTXs in both Q1 (the first quadrupore) and Q3 (the third quadrupore) [21], was used to quantify CTXs in fish specimens in the present study. We found that the most important MS parameters to obtain [M+Na]^+^ ions were DP (declustering potential) voltage and TEM (ion source temperature). Higher values of these parameters resulted in better signal-to-noise gains of the [M+Na]^+^ ions, due to the stable nature of sodium adduct ions under heating, and high DP where interfering ions are mostly broken down. LOD and LOQ of CTX-1B, obtained in our SIM LC/MS, were 0.09 and 0.32 ng/g flesh, respectively. Although these values are higher than a guidance level of 0.01 ng CTX-1B equivalent/g flesh, issued by the United States Food and Drug Administration (US FDA) [16], and inferior to those reported in previous [21,24] and the latest study [30], it was demonstrated that our SIM LC/MS method is useful for identification of causative toxins at clinically relevant levels. Determination of accurate masses of CTX analogues by TOF LC/MS could be very useful for identifications of CTX analogues, in the case where reference materials of toxins are not available.

Matrix effects suppressing the intensity of the [M+Na]^+^ ion of CTX-1B from a red snapper extract was confirmed in our SIM LC/MS method. Due to the shortage of CTX-1B reference material, the matrix effect was evaluated on a single point concentration of CTX-1B. However, this should be further evaluated by comparing the slope of calibration curves of CTX-1B in methanol and in the presence of the fish flesh matrix, as reported previously [31]. Further investigation of the matrix effects on our LC/MS method will be required when sufficient amounts of CTX analogues are available.

## 4. Materials and Methods

### 4.1. Specimens Collection

Red snapper specimens implicated in the CFP incidents (Table 1) were immediately sent to the Institute of Oceanography, Vietnam Academy of Science and Technology, and kept at −20 °C. Another red snapper specimen showing negative in the mouse bioassay (MBA) was also kept at −20 °C.

### 4.2. Reagents and Standards

HPLC- (acetonitrile, methanol) and analytical-grade solvents (acetone, methanol, hexane, diethyl ether, ethyl acetate) were purchased from Kanto Chemical Co., Inc. (Tokyo, Japan) and Fuji Film Wako Pure Chemical Co. (Osaka, Japan). Analytical-grade reagents (formic acid, ammonium formate) were purchased from Nacalai (Kyoto, Japan). Distilled water was passed through a Milli-Q water purification system (Millipore, Bedford, MA, USA) for the preparation of LC mobile phases. CTX-1B was kindly provided from Prof. Takeshi Yasumoto of Japan Food Research Laboratories. CTX-3C, CTX-4A and CTX-4B were kindly provided from Dr. Mireille Chinain of Institut Louis Malardé.

### 4.3. Genetic Analyses on the Nuclear 16S rDNA and Mit COX1

Whole genomic DNA was extracted from fish flesh by use of the QuickGene DNA tissue kit S (FUJIFILM, Tokyo, Japan), according to the manufacturer’s instructions. The mitochondrial 16S rRNA gene (16Sar and 16Sbr) and COI (FF2d and FR1d) were amplified according to previous methods [32,33]. We performed the PCRs in a reaction mixture (10 µL) containing ca. 50 ng of template DNA, 0.2 mM of each dNTP, 0.5 μM of each designed primer pair, 1× PCR buffer (10 mM Tris-HCl, pH 8.3, 500 mM KCl, 15 mM MgCl_2_, 0.01% *w/v* gelatin, Applied Biosystems, Foster City, CA, USA), and 0.25 U of Ampli *Taq* Gold (Applied Biosystems) on a thermal cycler (PC-808, ASTEC, Fukuoka, Japan) to amplify both regions. The PCR cycling conditions were as follows: 10 min at 94 °C, 38 cycles of 30 s at 94 °C, 30 s at 55 °C, and 45 s at 72 °C, and a final elongation for 5 min at 72 °C. The PCR products were transformed into DH5α cells (Promega, Madison, WI, USA) after ligation into the pGEM T-Easy Vector (Promega). The plasmid DNAs were purified after color selection. DNA sequences were determined using Dynamic ET terminator cycle sequencing kit (GE Healthcare, Little Chalfont, UK), in combination with M13 Reverse and U19 primers, and analyzed on a DNA sequencer (ABI3730, Applied Biosystems, Foster City, CA, USA).

The mitochondrial 16S rRNA gene and COI regions of the sample, excluding the primer regions, were aligned and edited manually using MEGA 7 [34]. Species identification was carried out using NCBI BLAST searching.

### 4.4. Mouse Bioassay

The fish specimen was extracted and tested according to the official mouse bioassay method used in Japan for CTXs [35]. Whole amounts of the fish flesh were extracted with acetone and the acetone extract was evaporated, then residual water extract was partitioned with diethyl ether. Diethyl ether layer was evaporated, and the residue was dissolved in methanol/distilled water (9:1, *v/v*). The aqueous methanol extract was partitioned with hexane to remove neutral lipids. The aqueous methanol extract was evaporated, and an aliquot was dissolved in 1% Tween 60 in normal saline solution with a concentration of 40 g fish flesh in 1 mL solution. A 1 mL portion was injected into each of three ddY (Deutschland, Denken and Yoken) or ICR (Institute of Cancer research) mice with 17–20 g body weight. Injected mice were observed for 24 h. When at least two mice died by injection of the testing solution, the toxicity was defined as 1 mouse unit (MU), which corresponds to 0.025 MU/g fish flesh, because 40 g fish flesh was used. Serial dilutions of the extract with 1% Tween 60 solution were prepared and tested in the same manner. When the most diluted extract did not kill mice or killed only one mouse within 24 h, this bioassay was concluded. Lethal time was not counted in this bioassay. The dilution factor was determined by the last diluted extract killing more than two of three mice within 24 h. As the result, the total toxicity was calculated by multiplication of the dilution factor to 0.025 MU/g.

### 4.5. Clean-Up of Fish Specimens for LC/MS

The clean-up of fish specimens was carried out according to a previous method [21]. The extracts corresponding to 5 g fish flesh were evaporated and cleaned-up with Florisil (InertSep FL-PR, 500 mg) followed by PSA (Pressure Swing Adsorption) (InterSep PSA, 200 mg). Triplicate analysis from the clean-up to LC/MS were carried out for each fish extract.

### 4.6. Quadrupole LC/MS

LC/MS analysis of toxins was carried with a model 1200 liquid chromatograph (Agilent, Palo Alto, CA, USA) coupled to a hybrid triple quadrupole/linear ion trap mass spectrometer Q Trap^TM^ 3200 (PE-SCIEX, Thornhill, ON, Canada). LC conditions were followed [20], but with modification of the column, flow rate, and gradient conditions to reduce high pressures to the LC pump [21]. Separation was performed on an Agilent Poroshell 120 EC-C18 (100 × 2.1 mm i.d., 2.7 mm particle size) column maintained at 20 °C. Eluent A was 5 mM ammonium formate and 0.1% formic acid in water, and B was methanol. A linear gradient elution, from 80% to 95% B, was performed over 10 min and then held for 10 min. The flow rate was 0.25 mL/min, and the injection volume was 5 µL. Instead of multiple reaction monitoring (MRM) selecting [M+Na]^+^ in both the target parent and the fragment ions in Q1 and Q3 [21], selected ion monitoring (SIM) selecting [M+Na]^+^ in Q1 with a dwell time of 63 ms for each analogue was applied [20]. Ion spray voltage (IS) and temperature in IS (TEM) were set at 5500 V and 500 °C. Ion source gas 1 (GS1) and GS2 were 80 and 30, respectively. Declustering potential (DP) and entrance potential (EP) were 400 and 12 V, respectively. Other MS parameters are shown in Table 4.

### 4.7. Matrix Effect on the Quadrupole SIM LC/MS for [M+Na]^+^ of CTX-1B

An MBA negative red snapper extract corresponding to 5 g fish flesh was evaporated and cleaned-up following the previously described protocol, then fortified with CTX-1B at 22 ppb concentration, corresponding to 0.88 ng/g fish flesh in our experimental procedure. Limits of detection (LOD) and quantification (LOQ) were also obtained from this sample. Signal-to-noise ratios S/N 3 and S/N 10 were defined as LOD and LOQ, respectively, calculated by Analyst Software 1.6.2 version.

### 4.8. Quadrupole Time of Flight (QTOF) LC-MS/MS

QTOF LC-MS/MS analysis of toxins was carried with a model Ultimate 3000 liquid chromatograph (Thermo Dionex, Sunnyvale, CA, USA) coupled to a micrOTOF-Q II mass spectrometer (Bruker Daltonics, Bremen, Germany) equipped with an ESI source. LC conditions were basically the same as those for quadrupole LC/MS described in the present study. Full scan analysis was performed with *m*/*z* range of 400 and 1800, to obtain elemental formulae of CTX analogues. ESI conditions were capillary voltage, 4500 V; dry heater, 200 °C; dry gas flow, 8.0 L/min; nebulizer gas, 1.6 bar; quadrupole ion energy 5.0 eV, collision energy 10.0 eV, collision cell RF, 1000 Vpp. Smart Formula software of Data Analysis 4.0 SP2 (Bruker Daltonics, Bremen, Germany) was used for elemental formula analysis.

## Figures and Tables

**Table 1:** Summary of implicated fish and symptoms associated with ciguatera fish poisoning (CFP) incidents in Vietnam in 2014 and 2016.

Incident	Implicated Fish	Origin of Implicated Fish	Location of Incident	Date of Incident	Number of Patients/Number of Consumers
1	Red snapper *Lutjanus bohar* *^1^ (approx. 5 kg body weight)	Phan Thiet City, Binh Thuan Province	Ho Chi Minh City	17 July 2014	6/6 (family members)
	**Symptoms:** Developed 3–4 h after eating: Dizziness, headache, pain on muscle and junction joints, nausea, vomiting, and diarrhea for a week.				
2	Red snapper *Lutjanus bohar* (approx. 2 kg body weight)	Nha Trang City, Khanh Hoa Province	Nha Trang City, Khanh Hoa Province	22 June 2016	4/4 (family members)
	**Symptoms:** Developed 1–2 h after eating: Pain in muscles, joints and the back head continuously for several days. After hospitalization for 3 days, the patients came back home but one of them (father) had to be hospitalized again for one more week as symptoms persisted. The father said he felt there was a cold wind blowing into the mouth when drinking water.				

*^1^ Identification of fish species was carried out by 16S and COX gene analyses.

**Table 2:** Accurate masses of observed ions of ciguatoxins detected in red snappers in Vietnam.

Sample		CTX-1B			54-deoxy CTX-1B			52*-epi*-54-deoxyCTX-1B		
	Ions	[M+NH_4_]^+^	[M+Na]^+^	[M+K]^+^	[M+NH_4_]^+^	[M+Na]^+^	[M+K]^+^	[M+NH_4_]^+^	[M+Na]^+^	[M+K]^+^
	Formula	C_60_H_90_N_1_O_19_	C_60_H_86_O_19_Na	C_60_H_86_O_19_K	C_60_H_90_N_1_O_18_	C_60_H_86_O_18_Na	C_60_H_86_O_18_K	C_60_H_90_N_1_O_18_	C_60_H_86_O_18_Na	C_60_H_86_O_18_K
	Theoretical Values	1128.6102	1133.5656	1149.5395	1112.6152	1117.5706	1133.5446	1112.6152	1117.5706	1133.5446
Authentic CTX-1B		1128.6074 (2.4) ^*1^	1133.5626 (2.6)	1149.5345 (4.3)	-	-	-	-	-	-
Fish flesh from incident 1		1128.6137 (−3.1)	1133.5688 (−2.9)	1149.5497 (−8.9)	1112.6185 (−2.9)	ND	ND	1112.6091 (5.5)	ND	ND
Fish viscera from incident 2		1128.6070 (2.8)	ND ^*2^	ND	1112.6129 (2.1)	ND	ND	1112.6073 (7.1)	ND	ND
Fish flesh from incident 2		1128.6059 (3.8)	1133.5623 (2.9)	1149.5359 (3.1)	1112.6141 (1.0)	ND	ND	1112.6142 (0.9)	1117.5734 (−2.5)	ND

*^1^ Measured values (error ppm). *^2^ ND: Not detected.

**Table 3:** Ciguatoxin contents in *Lutjanus bohar* implicated in CFP in Vietnam in 2014 and 2016.

Incident	Body Part	CTX Contents (ng/g) *^1^			Total Toxicity (MU/g) Estimated by LC/MS *^4^	Toxicity (MU/g) by MBA
		CTX-1B Average (SD)	54-deoxyCTX-1B Average (SD)	52*-epi*-54-deoxyCTX-1B Average (SD)		
1	flesh	3.67 (0.21)	0.36 (0.18) *^2^	0.74 (0.24) *^2^	0.58	0.15
2	viscera	0.92 (0.03)	ND	ND *^3^	0.13	-
2	flesh	2.77 (0.05)	ND	ND *^3^	0.40	0.35–0.40

*^1^ Data was obtained by triplicate analyses; *^2^ Quantification of deoxy-CTX-1B was carried out by using the peak area of a CTX-1B standard; *^3^ Trace levels of toxin were detected by TOF LC/MS; *^4^ Total toxicity (MU/g) = CTX-1B content (ng/g)/7 (ng/MU) + 52-*epi*-54-deoxyCTX-1B (ng/g)/14 (ng/MU); Conversion factors (ng/MU) of CTX-1B and 52-*epi*-54-deoxyCTX-1B are 7 and 14, respectively [13,14,22,23].

**Table 4:** MS parameters on the SIM for detection of CTX analogues.

Compound	[M+Na]^+^ *m/z*	CEP (Volts)
CTX-1B(P-CTX-1)	1133.6	42
52-*epi*-54-deoxyCTX-1B (P-CTX-2)	1117.6	41
54-deoxyCTX-1B (P-CTX-3)	1117.6	41
CTX-4A	1083.6	40
CTX-4B	1083.6	40
M-seco-CTX-4A/B	1101.6	41
CTX-3C	1045.6	39
49-*epi*-CTX-3C	1045.6	39
51-hydroxyCTX-3C	1061.6	40
2-hydroxyCTX-3C	1063.6	40
2,3-dihydroxyCTX-3C	1079.6	40
M-seco-CTX-3C	1063.6	40
M-seco-CTX-3C methyl acetal	1077.6	40
gambierol	779.5	32
gambieric acid A	1101.6 *^1^	41
gambieric acid B	1115.6 *^1^	41

CEP: collision cell entrance potential; *^1^ [M+2Na−H]^+^.

## References

[1] Food and Agriculture Organization (FAO) (2004). Marine Biotoxins.

[2] Lewis R.J. (2001). The changing face of ciguatera. Toxicon.

[3] Vilariño N., Louzao M.C., Abal P., Cagide E., Carrera C., Vieytes M.R., Botana L.M. (2018). Human poisoning from marine toxins: Unknowns for optimal consumer protection. Toxins.

[4] Adachi R., Fukuyo Y. (1979). The thecal structure of a marine toxic dinoflagellate Gambierdiscus toxicus gen. et sp. nov. collected in a ciguatera-endemic area. Bull. Jpn. Soc. Sci. Fish..

[5] Yasumoto T., Nakajima I., Bagnis R., Adachi R. (1977). Finding of a dinoflagellate as a likely culprit of ciguatera. Bull. Jpn. Soc. Sci. Fish..

[6] Bagnis R., Chanteau S., Chungue E., Hurtel J.M., Yasumoto T., Inoue A. (1980). Origins of ciguatera fish poisoning: A new dinoflagellate, Gambierdiscus toxicus Adachi and Fukuyo, definitively involved as a causal agent. Toxicon.

[7] Chinain M., Darius H.T., Ung A., Cruchet P., Wang Z., Ponton D., Laurent D., Pauillac S. (2010). Growth and toxin production in the ciguatera-causing dinoflagellate *Gambierdiscus polynesiensis* (Dinophyceae) in culture. Toxicon.

[8] Litaker R.W., Holland W.C., Hardison D.R., Pisapia F., Hess P., Steven R., Kibler S.R., Tester P.A. (2017). Ciguatoxicity of Gambierdiscus and Fukuyoa species from the Caribbean and Gulf of Mexico. PLoS ONE.

[9] Pisapia F., William C., Holland W.C., Hardison D.R., Litaker R.W., Santiago Fraga S., Nishimura T., Masao Adachi M., Lam Nguyen N.L., Séchet V. (2017). Toxicity screening of 13 Gambierdiscus strains using neuro-2a and erythrocyte lysis bioassays. Harmful Algae.

[10] Silva M., Rodriguez I., Barreiro A., Kaufmann M., Neto A.I., Hassouani M., Sabour B., Alfonso A., Botana L., Vasconcelos V. (2015). First Report of Ciguatoxins in Two Starfish Species: *Ophidiaster ophidianus* and *Marthasterias glacialis*. Toxins.

[11] Roué M., Darius H.T., Picot S., Ung A., Viallon J., Gaertner-Mazouni N., Sibat M., Amzil Z., Chinain M. (2016). Evidence of the bioaccumulation of ciguatoxins in giant clams (*Tridacna maxima*) exposed to Gambierdiscus spp. cells. Harmful Algae.

[12] Darius H.T., Roue M., Sibat M., Viallon J., Gatti C.M., Vandersea M.W., Tester P.A., Litaker R.W., Amzil Z., Hess P. (2018). *Tectus niloticus* (Tegulidae, Gastropod) as a Novel Vector of Ciguatera Poisoning: Detection of Pacific Ciguatoxins in Toxic Samples from Nuku Hiva Island (French Polynesia). Toxins.

[13] Murata M., Legrand A.M., Ishibashi Y., Yasumoto T. (1989). Structures of ciguatoxin and its congener. J. Am. Chem. Soc..

[14] Yasumoto T., Igarashi T., Legrand A.M., Cruchet P., Chinain M., Fujita T., Naoki H. (2000). Structural elucidation of ciguatoxin congeners by fast-atom bombardment tandem mass spectroscopy. J. Am. Chem. Soc..

[15] Lewis R.J., Vernoux J.P., Brereton I.M. (1989). Structure of *Caribbean ciguatoxin* isolated from *Caranx latus*. J. Am. Chem. Soc..

[16] Dickey R.W., Plakas S.M. (2010). Ciguatera: A public health perspective. Toxicon.

[17] Chan T.Y.K. (2015). Ciguatera Fish Poisoning in East Asia and Southeast Asia. Mar. Drugs.

[18] Dao V.H., Pham X.Y. Ciguatera Fish Poisoning in Vietnam. http://file.iocwestpac.org/TMO/reference%20materials/CFP%20in%20Vietnam.pdf.

[19] Ho V.T., Nguyen N.L., Doan N.H. (2010). Benthic Dinoflagellates in Vietnamese Waters.

[20] Suzuki T., Dao V.H., Uesugi A., Uchida H. (2017). Analytical challenges to ciguatoxins. Curr. Opin. Food Sci..

[21] Yogi K., Oshiro N., Inafuku Y., Hirama M., Yasumoto T. (2011). Detailed LC-MS/MS analysis of ciguatoxins revealing distinct regional and species characteristics in fish and causative alga from the Pacific. Anal. Chem..

[22] Satake M., Morohashi A., Oguri H., Oishi T., Hirama M., Harada N., Yasumoto T. (1997). The Absolute Configuration of Ciguatoxin. J. Am. Chem. Soc..

[23] Yasumoto T. (2001). The chemistry and biological function of natural marine toxins. Chem. Rec..

[24] Stewart I., Geoffrey K.E., Poole S., Graham G., Paulo C., Wickramasinghe W., Sadler R., Shaw G.R. (2010). Establishing a public health analytical service based on chemical methods for detecting and quantifying Pacific ciguatoxin in fish samples. Toxicon.

[25] Mak Y.L., Wu J.J., Chan W.H., Murphy M.B., Lam C.W., Chan L.L., Lam P.K.S. (2013). Simultaneous quantification of Pacific ciguatoxins in fish blood using liquid chromatography–tandem mass spectrometry. Anal Bioanal. Chem..

[26] Wong C.K., Hung P., Lo J.Y. (2014). Ciguatera fish poisoning in Hong Kong—A 10-year perspective on the class of ciguatoxins. Toxicon.

[27] European Food Safety Authority (EFSA) (2010). Scientific Opinion on marine biotoxins in shellfish—Emerging toxins: Ciguatoxin group. EFSA J..

[28] Lehane L., Lewis R.J. (2000). Ciguatera: Recent advances but the risk remains. Int. J. Food Microbiol..

[29] Yasumoto T. (2005). Chemistry, etiology, and food chain dynamics of marine toxins. Proc. Jpn. Acad. Ser. B Phys. Biol. Sci..

[30] Sibat M., Herrenknecht C., Darius H.T., Roue M., Chinain M., Hess P. (2018). Detection of pacific ciguatoxins using liquid chromatography coupled to either low or high resolution mass spectrometry (LC-MS/MS). J. Chromatogr. A.

[31] Fux E., Rode D., Birea R., Hess P. (2008). Approaches to the evaluation of matrix effects in the liquid chromatography-mass spectrometry (LC-MS) analysis of three regulated lipophilic toxin groups in mussel matrix (*Mytilus edulis*). Food Addit. Contam..

[32] Natalia V.I., Zemlak T.S., Hanner R.H., Hebbert P.D.N. (2016). Universal primer cocktails for fish DNA barcoding. Mol. Ecol. Resourc..

[33] Palumbi S.R., Hillis D.M., Moritz C., Mable B.K. (1996). Nucleic acids II: The polymerase chain reaction. Molecular Systematics.

[34] Kumar S., Stecher G., Tamura K. (2016). MEGA7: Molecular evolutionary genetics analysis version 7.0 for bigger datasets. Mol. Biol. Evol..

[35] Oshiro N., Kentaro Y., Asato S., Sasaki T., Tamanaha K., Hirama M., Yasumoto T., Inafuku I. (2010). Ciguatera incidence and fish toxicity in Okinawa, Japan. Toxicon.

